# Classification of Hydration in Clinical Conditions: Indirect and Direct Approaches Using Bioimpedance

**DOI:** 10.3390/nu11040809

**Published:** 2019-04-10

**Authors:** Henry C. Lukaski, Nicanor Vega Diaz, Antonio Talluri, Lexa Nescolarde

**Affiliations:** 1 Department of Kinesiology and Public Health Education, University of North Dakota, Grand Forks, ND 58202-7166, USA; henry.lukaski@und.edu; 2 Nephrology Service, University Hospital of Grand Canary and Faculty of Science, University Los Palmas, 35019 Los Palmas, Grand Canary, Spain; nvegdia@gobiernodecanarias.org; 3 Antonio Talluri, Fatbyte, Inc., 50012 Bagno a Ripoli, Florence, Italy; info@fatbytesrl.com; 4 Department of Electronic Engineering, Universitat Politècnica de Catalunya, 08034 Barcelona, Spain

**Keywords:** fluid overload, resistance, reactance, bioelectrical impedance vector analysis, bioelectrical impedance spectroscopy, malnutrition

## Abstract

Although the need to assess hydration is well recognized, laboratory tests and clinical impressions are impractical and lack sensitivity, respectively, to be clinically meaningful. Different approaches use bioelectrical impedance measurements to overcome some of these limitations and aid in the classification of hydration status. One indirect approach utilizes single or multiple frequency bioimpedance in regression equations and theoretical models, respectively, with anthropometric measurements to predict fluid volumes (bioelectrical impedance spectroscopy—BIS) and estimate fluid overload based on the deviation of calculated to reference extracellular fluid volume. Alternatively, bioimpedance vector analysis (BIVA) uses direct phase-sensitive measurements of resistance and reactance, measured at 50 kHz, normalized for standing height, then plotted on a bivariate graph, resulting in a vector with length related to fluid content, and direction with phase angle that indexes hydration status. Comparison with healthy population norms enables BIVA to classify (normal, under-, and over-) and rank (change relative to pre-treatment) hydration independent of body weight. Each approach has wide-ranging uses in evaluation and management of clinical groups with over-hydration with an evolving emphasis on prognosis. This review discusses the advantages and limitations of BIS and BIVA for hydration assessment with comments on future applications.

## 1. Introduction

Routine assessment of hydration status to support patient care persists as a challenge. The preponderance of methods suffers from impracticality and insensitivity [1,2]. Historically, hydration assessment was synonymous with the use of tracer dilution methods to estimate total body water (TBW), extracellular water (ECW), and plasma volume (PV) with the calculation of intracellular water (ICW, ICW = TBW − ECW). This approach is invasive, time-consuming, costly, and prone to ambiguous interpretations of hydration status because of reliance on the assumption of constant TBW to body weight (% TBW), which is prejudiced by inter-individual differences in body composition (adipose, muscle mass, or cachexia)**,** or TBW to fat-free mass (FFM) (% TBW/FFM) that requires an independent determination of FFM [3,4,5,6]. Thus, the measurement of fluid volumes to assess human hydration status has limited value.

Laboratory tests of plasma or serum constituents (osmolality, sodium, natriuretic peptide, and other promising proteins), urine characteristics (osmolality, specific gravity, conductivity, and 24-h output), and saliva and tear composition (osmolality) have inadequate feasibility and lack sensitivity in a clinical setting [2]. Imaging techniques (radiography and ultrasound) and invasive procedures (catherization) are costly and fail to identify early disturbances in hydration. Simple assessments such as self-reported thirst, patient history, and physical examination are subjective and lack sensitivity [2,7]. Thus, the need persists for a method that is non-invasive, valid (accurate and sensitive), practical, cost-effective, and provides real-time discrimination within the spectrum of the hydration status. Classification of hydration as normal, less, or greater than normal is a practical and valid approach that can overcome these limitations. Burgeoning evidence supports bioelectrical impedance as a unique and promising method to classify the hydration status of an individual.

Bioimpedance (BI) is the general term describing the safe, non-invasive measurement of the passive electrical characteristics of an organism after introduction of a painless, low-level alternating current into the body [8,9,10]. Bioimpedance analysis (BIA), however, augments BI measurements with theoretical biophysical models and statistical (regression) equations contributing to “black box” predictions of fluid volumes that can be unreliable and imprecise. Bioimpedance per se offers direct, uncomplicated measurements that facilitate a practical and valid approach to monitor hydration.

This review emphasizes the use of BI to classify hydration. It describes the biophysical basis of the BI method, summarizes different technical approaches and their limitations to estimate fluid volumes, discusses advantages of BI measurements compared to calculated fluid volumes, highlights clinical applications of BI emphasizing its emerging role in prognosis, and provides recommendations to improve and expand the use of BI in clinical applications.

## 2. Bioelectrical Impedance

### 2.1. Bioimpedance Basics 

**Figure 1:**
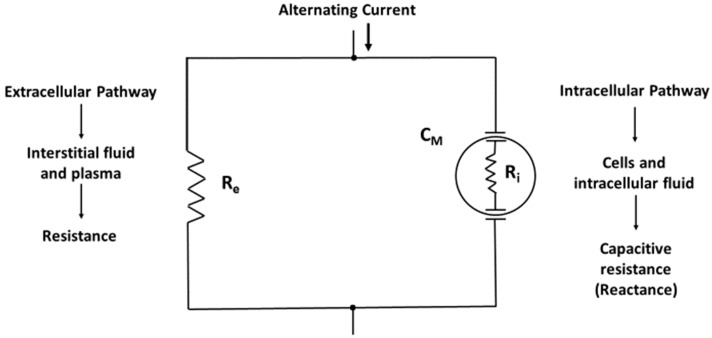
Illustration of the body as a network of resistors and capacitors in a parallel configuration. The alternating current usually exceeds 1 kHz and typically is 50 kHz. C_M_ is membrane capacitance and R_e_ and R_i_ is extracellular and intracellular resistance, respectively.

**Figure 2:**
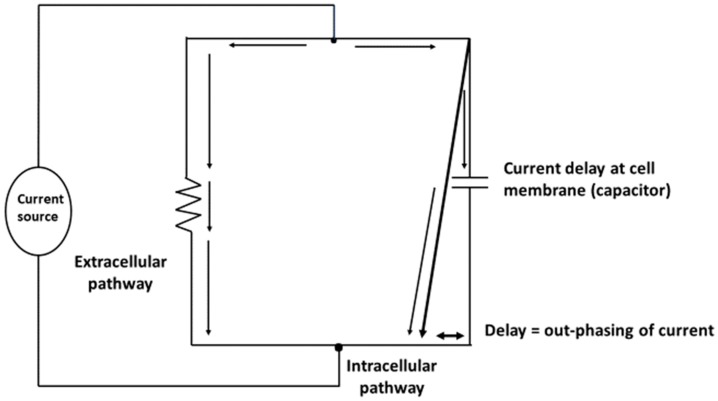
Representation of the body as a parallel resistor-capacitor (RC) equivalent circuit. Delay of the current penetration at the cell membrane causes an out-phasing of current.

The physical basis of the BI method is the awareness that the human body is a network of resistors and capacitors [8]. Physiological fluids behave as resistors and cell membranes act as capacitors (Figure 1). Thus, the body may be represented as a parallel resistor-capacitor (RC) equivalent circuit (Figure 2) in which the introduced alternating current divides into resistive (fluid and electrolytes) and capacitive (cell membranes and tissue interfaces) pathways. The lower specific resistivity of body fluids and tissues containing water and electrolytes compared to intact lipid-laden cell membranes [11] enables frequency-dependent impedance measurements [12]. A low-level alternating current continuously passes predominantly through the resistive component but concurrently delayed (temporarily stored) by capacitive elements in tissues and tissue interfaces. Importantly, a variable amount of very low-frequency current, regardless at which frequency the current is introduced, can penetrate the membranes of muscle cells, particularly when the current is parallel to the muscle fiber [13]. Cell membranes are capacitive elements surrounding the intracellular fluid (ICF) in a series circuit that exists in parallel with the water-containing interstitial gel or extracellular fluid (ECF). Any alternating current will penetrate the capacitive component (reactance, Xc) of the cell membrane in proportion to the frequency of the applied current. Reactance is inversely related to frequency (f) and capacitance (C, Farads): Xc (ohm, Ω) = 1/[(2π • f • C)]. Capacitance is the ability of a system or circuit to store an electrical charge.

At low frequencies (e.g., 1–5 kHz), alternating current flows largely in the ECF but not in a direct or fixed proportion relative to the ICF. Similarly, at 50 kHz alternating current does not distribute in proportion to fluid distribution (extra- to intra-cellular fluid volume) but relative to capacitive elements. Thus, the phase angle (PhA) is frequency-dependent, principally due to the amount of Xc, and is an index of the amount of applied current that penetrates the capacitive element or cell membranes. Current that is restricted or delayed at (intact) cell membranes becomes out-phased from the voltage drop that occurs at the cell.

**Figure 3:**
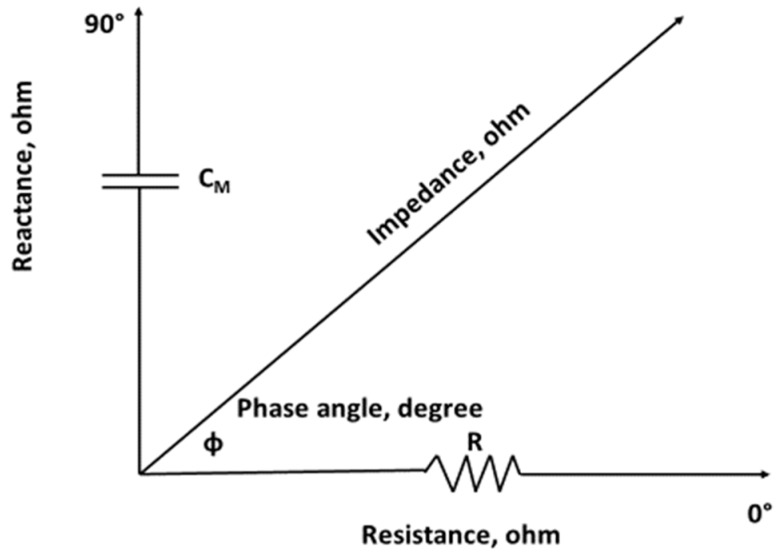
Geometric relationships among the resistance, reactance (capacitance, C_M_), impedance, and phase angle.

Impedance (Z) is the broad term describing the opposition to the flow of alternating current by any biological conductor, and is defined by the type of electrical current introduced to a circuit. When direct current is applied, the total conductor is termed resistance (R) whereas when alternating current is introduced, it is called Z. Direct current passes only through resistive elements but alternating current flows through resistive and capacitive elements. The resistive (R) component of Z is independent of frequency so it has the same measurement value (Ω) when either direct or alternating current is used. The capacitor in a biological circuit serves as an insulator and may be envisioned as an imaginary component of Z when direct current is introduced. However, when alternating current is present, the imaginary component acts as a poor conductor with capacitive resistance and is termed reactance (Xc), which is frequency-dependent. Thus, Z is a complex number, Z^2^ = R^2^ + Xc^2^, and characterizes the specific fluid and cellular components of an organism. Impedance is a vector with length and position on the bivariate RXc plot (Figure 3). The PhA describes the position of Z and is the angular transformation of Xc to R [arc tangent (Xc/R) • (180°/*π*)] expressed in radian degrees. 

### 2.2. Measurement of Bioelectrical Impedance

Assessment of whole-body hydration uses surface, gel (silver-silver chloride) or modern hydrogel electrodes, and tetrapolar electrode placements. Whole-body BI measurements employ traditional limb placements with paired current-introducing (source) and voltage-drop-sensing (detector) electrodes placed on the distal wrist and ankle [8,10]. 

## 3. Volume Quantification in Hydration Assessment: Limitations and Imprecision

A primary factor for reliable hydration assessment is the technical validity of the BI measurement. It includes the accuracy of the instrument, which is determined with a precision (<1%) circuit of a resistor and a capacitor in a parallel circuit, and precision or reproducibility that requires repeated measurements of a validation circuit and repetitive in vivo measurements. The technical validity should be <2% for all BI measurements [9]. Similarly, the validity of the reference tracer dilution methods must be assessed with technical accuracy and precision determined to be <2%. Inter-individual biological sources of error for the tracer dilution methods, including hydrogen exchange and anion (bromine-chlorine) shifts that are >4% [14,15], directly affect the variability of fluid volume estimates. 

### 3.1. Single-Frequency Bioimpedance

Clinical investigators applied different BI approaches to estimate fluid volumes as indicators of hydration. They mostly used phase-sensitive single-frequency (50 kHz) BI because of the high signal-to-noise ratio [9,16] or multiple-frequency (5 kHz to 1 MHz) measurements coupled with either multiple regression equations or theoretical biophysical models to predict fluid volumes [10,17,18,19]. Each of these approaches has notable concerns that limit their general application in the estimation of fluid volumes. The electrical volume model adapted from Ohm’s Law is the physical basis for estimation of fluid volumes with single- and multiple-frequency BI measurements [10]. The volume (V) of a conductor (fluid plus electrolytes) depends on the length (L) and cross-sectional area (A) of a cylindrical conductor with constant geometry and composition (specific resistivity or ρ), so that V = ρ(L^2^/R). Standing height (Ht) is a biological surrogate for L, so V = ρ(Ht^2^/R). The assumptions of this model, which include the body is a uniform cylinder of constant chemical composition (e.g., water content) of the conductor, are open to criticism [18]. Specifically, the body consists of five cylinders (two arms, two legs, and the thorax) with different geometry (varying length and diameter) and different chemical composition (specific resistivities of different tissues) including a disputed constant hydration of the fat-free body (73%). Researchers exploited this bioelectrical volume model, together with various anthropometric information (Ht, weight (Wt), age, and gender), to develop and validate multiple regression prediction equations using single- and multiple-frequency measurements for TBW estimates in healthy and ill adults [18]. 

A technical issue exists with any application of these regression models. The independent variable is R and not Z. Thus, only phase-sensitive BI instruments should be used in the application of specific single-frequency prediction models using R as an independent variable. Although Z and R are highly correlated (R^2^ > 0.9), the magnitude of Z is greater than R (1% to 2%) because Z includes the value of Xc, which is not negligible (~10% of R) and includes biologically important information for hydration classification [9,10]. Similarly, predictions based on multi-frequency BI measurements should ensure that appropriate devices are used to obtain the required Z or R measurements.

Critical evaluation of a population-based model to predict TBW highlights a practical limitation to the use of BIA to estimate the fluid volume. Sun et al. [20] derived and validated TBW prediction equations in a large sample of healthy adults of diverse ethnicity:

Males: TBW (L) = 0.87 + 0.43 (Ht^2^/R) + 0.20 Wt

Females: TBW (L) = 3.27 + 0.45 (Ht^2^/R) + 0.12 Wt

Cross-validation of these equations revealed a large predictive error (standard error of estimate (SEE)) of 3.8 L and 2.6 L, for the males and females, respectively. The magnitude of these prediction errors is too large (e.g., imprecise) to estimate TBW or to identify change in TBW of an individual and, therefore, limits these equations to observational or epidemiological studies.

### 3.2. Bioelectrical Impedance Spectroscopy (BIS)

Some BI instruments measure Z over a wide range of frequencies (e.g., 5 kHz up to 1 MHz), report various measurements and then use programmed software to estimate fluid volumes [17]. Certain non-phase-sensitive devices only measure Z and provide the ratio of Z at low and high frequencies (Z_low_/Z_high_) that is equivalent to PhA at 50 kHz. Similarly, they also use the Z_low_/Z_high_ to compute the phase and cannot perform valid calculations to form the impedance modulus for the Cole plot.

The BIS method relies on the assumption that low-frequency current flows through the ECF and high-frequency current penetrates ECF and ICF. These assumptions are open to criticism largely because they were derived from in vitro studies of cells suspended in fluid and ignore the cell-cell interfaces that occur in tissues [10]. Additionally, investigators agree that at the lowest frequencies some current penetrates cell membranes (e.g., extracellular and intracellular paths) and that at the highest frequencies current does cross all cells (e.g., extracellular and intracellular paths) [11,21,22,23].

**Figure 4:**
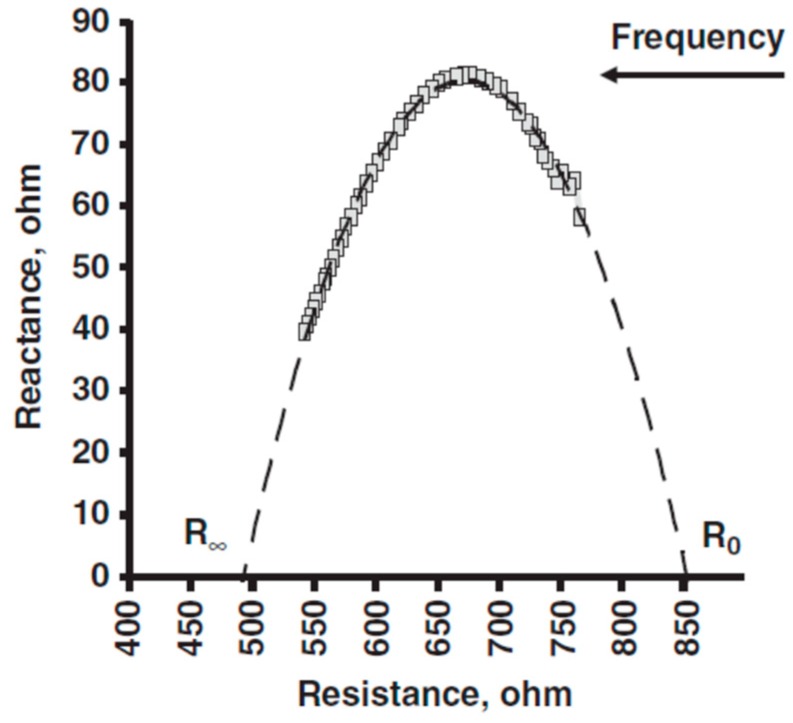
Plot of reactance and resistance of a healthy male obtained with a Xitron 4200 and derived using non-linear curve-fitting software based on the Cole model. Note that the majority of values (dashed lines) were estimated. R_0_ and R_∞_ were calculated and they approximate resistance at 0 and the highest frequency, respectively.

The BIS method uses non-linear least-square (polynomial) modeling of limited phase-sensitive multi-frequency measurements of Z and Xc, calculated from PhA, then extrapolates these values to generate a Cole plot (Figure 4). This mathematical model [24] calculates theoretical resistivity values that are used to approximate fluid volumes. The extrapolated (modeled) variables are R_0_, also termed as Re (the resistance of the extracellular fluid or interstitial resistivity), and R_∞_ (resistance associated with the sum of extracellular and intracellular fluids), critical frequency (f_C_) or the frequency at which Xc is maximal, and membrane capacitance (Cm). The value of calculated Cm depends on another fitted variable, time delay (Td), which represents the effect of cell membrane and orientation on the current transmission in the body. These resistance parameters are used to calculate intracellular resistance (Ri) as 1/Ri = 1/R∞ − 1/Re. 

Mixture theory uses these calculated resistance parameters with anthropometric measurements and some wide-ranging assumptions to estimate fluid volumes [17,25,26]. It relies on a theoretical model that estimates apparent conductivity in a heterogeneous entity composed of conductive (water and ions) and non-conductive (anhydrous materials such as bone, fat, and cell membranes) components such as the body. Fluid volumes, ECW, and ICW are predicted separately and summed to obtain the TBW. Calculation of the ECW and ICW depends on the estimated Cole resistance parameters, body scalar factors calculated from the Ht and Wt of an individual, an assumed body density value derived from body mass index (BMI), empirically derived gender-specific resistivity values, and other assumptions. Extracellular fluid volume (V_ECF_) is calculated as V_ECF_ = k_ECF_ • (Ht^2^ • Wt^0.5^/Re)^2/3^ with k_ECF_ = 10^-3^ • (K_B_^2^ • ρ_ECF_^2^/D_b_)^1/3^, where K_B_ is a body geometry factor that relates relative volume of the legs, arms, and trunk, ρ_ECF_ is the resistivity of the extracellular fluid, and D_b_ is total body density. Intracellular fluid volume (V_ICF_) also is calculated from the derived Cole resistance variables and gender-dependent resistivities of the ECW and ICW. These calculations are performed with software provided by the manufacturers of the different BIS instruments, and are subject to change.

Uncertain basic assumptions and constants of the original BIS approach [26,27,28,29] resulted in errors in the predictions of fluid volumes. Specifically, significant errors in the estimation of TBW (2 L) and ECW (~1 L) in individuals with increasing adiposity, characterized using BMI, were explained as the effect of increasing adipose tissue on the assumed resistivity of the ECW [30,31,32]. A proposed remedy to this limitation was the use of BMI as a proxy for adiposity [33]. 

A multi-center validation trial [33] of this revised BIS approach included healthy adults and dialysis patients and found that this change improved the sensitivity of the revised ECW predictions by decreasing the variability of the ECW estimate by 24% (0.6 L) in healthy and dialysis patients and reducing the variability of the TBW prediction in adults with BMI values of less than 20 and greater than 30 kg/m^2^. Although no significant differences between tracer dilution reference measurements and BIS predictions of the TBW, ECW, and ICW were found, there were wide limits of agreement (95% confidence intervals—95% CI). Specifically, the mean differences were small with wide limits of agreement for prediction of the TBW (−0.2 L (95% CI: 4.6 L)), ECW (−0.4 L; 95% CI: 2.9 L)), and ICW (0.2 L (95% CI: 4.1 L)). A limitation of the use of BMI as a surrogate for body fatness is the insensitivity of BMI to reliably differentiate the body composition (body fat and muscle mass) of an individual, healthy or ill [34]. These wide limits of agreement contribute to poor sensitivity of the ECW and TBW estimates estimated with BIS for individuals. 

### 3.3. Comparison of Fluid Volumes Estimated with Single-Frequency Bioimpedance and Bioelectrical Impedance Spectroscopy

Although a common opinion is that BIS, compared to a single-frequency BIA, provides more accurate estimates of fluid volume [23], experimental data fail to confirm this supposition. Impedance predictions of the TBW and ICW were equally accurate in comparison to isotope dilution estimates but included a significant bias (error) in single-frequency and BIS prediction of the ECW in steady-state hemodialysis patients [35]. Both impedance methods had a proportional error in estimation of the ICW largely due to assumptions of the BIS model and the single frequency equation used to predict the ICW with calculations from total body potassium, a specific marker for body cell mass. Similar findings came from another study of hemodialysis patients [36]. A consistent observation is the wide limits of agreement between the impedance and reference methods that cautions against the use of these methods for individual assessment of fluid volumes [37].

Other research supports these findings. In adults receiving growth hormone replacement therapy, the TBW was determined using isotope dilution and predicted from BI measurements, collected with the same multi-frequency instrument, using 50 kHz values and four published equations and the Hanai mixture [25] model with and without adjustment for BMI [38]. Measured and predicted TBW values were significantly and similarly correlated for linearity and concordance (r > 0.9). Comparisons of measured and predicted TBW values revealed limits of agreement with slightly greater variability (0.9 L to 2.7 L or 4% to 7%) for the 50 kHz predictions compared to variability estimates (0.6 L to 0.9 L or 1% to 2%) from the BIS predictions. Thus, both the single frequency prediction equations and BIS models performed equally well in predicting the TBW at the population level.

Follow up analyses determined the comparability of these different approaches to estimate the TBW at the level of an individual [38]. The mean absolute error of BIS was slightly improved compared to multiple regression equations using 50 kHz values (5% vs. 7% or 2 L vs. 3 L). These findings indicate that the precision of these BIA approaches may be appropriate for group comparisons but is too large for monitoring changes in the TBW in clinical conditions.

Any comparison of BIA-predicted fluid volumes with tracer dilution reference measurements suffers from technical and biological limitations. Sources of error include the validity (accuracy and precision) of the impedance measurement, error of prediction from the regression equation, intrinsic error of the reference method (technical accuracy and reproducibility, biological errors in assumptions of the dilution method), electrical-volume errors (anisotropy and body geometry), and biological variability (inter-individual differences in the diameter of body segments, limb lengths, and variation in body fatness) [37]. Use of the Cole model depends on the reliability of the fitting of the impedance data. Review of recent BIS publications reveals that individual subjects are removed from analyses because of failure to achieve acceptable tolerances (>99% reproducibility) of the repeated time delay estimates derived from curve fitting [35,36]. This observation indicates significant imprecision in the calculation of the current delay at membranes. Finally, use of different single-frequency mathematical prediction equations complicates comparisons. As suggested by Seone et al. [38,39], volume prediction models that include more independent variables than Ht^2^/R and weight tend to produce greater errors in estimated TBW that indicates a specificity of that regression equation and, thus, excludes its application in any sample or individual differing in characteristics from the original sample in which the regression model was developed. These factors as well as the imprecision of the prediction models, single-frequency and BIS, impedes their use in point-of-care individual assessments of hydration.

## 4. Classification of Hydration with Bioelectrical Impedance Vector Analysis (BIVA)

**Figure 5:**
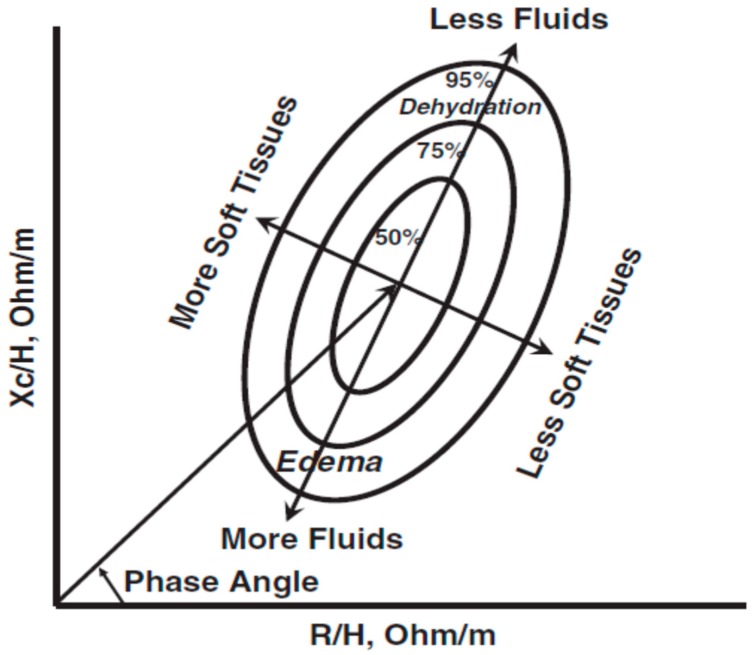
Resistance-reactance (RXc) plot with tolerance ellipses from healthy Caucasian males.

In contrast to the use of mathematical modeling of limited BI measurements, theoretical and regression prediction models, classification of hydration only requires precise BI measurements and reference values [40]. A 50 kHz phase-sensitive BI instrument directly measures R and Xc, which are normalized for standing height to achieve a standard resistivity and plotted on the RXc graph (Figure 5). The vector (Z) has a length that is inversely related to TBW and its position, described as the PhA, and provides information on tissue hydration. 

Bioelectrical impedance vector analysis (BIVA) enables classification (under-, normal, and over-hydration) and ranking of change in hydration (more or less than before treatment), as well as classification (more or less) of soft tissue mass by comparing vector position of an individual or a group to a healthy ethnicity-, age-, and gender-matched population [40]. Implementation of BIVA follows the work of Moore and Boyden [3] in which hydration is a function of fluid distribution in the extracellular and intracellular spaces, notably BCM. Clinically, over-hydration is expressed as an expansion of the extracellular fluid (interstitial fluid plus plasma), also termed fluid overload, relative to the BCM (intracellular fluid); patients with renal disease and heart failure are characterized as over-hydrated. Malnutrition, including cachexia, also is associated with an increase in ECW and a decrease in BCM. Hypo-hydration is seen with a decrease in ECW and little or no change in BCM. It can be acute as with excess fluid removal therapy or short-term with diseases causing excessive fluid loss (diarrhea or emesis).

Compared to single-frequency BIA and BIS predictions of fluid volumes, BIVA only has minimal error associated with BI measurement and reproducibility (1–2%) whereas fluid volume predictions include additional sources of error including regression error of the prediction equation (~10%), technical error in the reference method (~4%), limitations of the bioelectrical volume model (i.e., anisotropy of tissues and geometry), and biological variability (i.e., inter-individual body composition differences) that propagate. Therefore, for an individual, classification and ranking of hydration is more precise and accurate than is the quantification of fluid volume because BIVA is independent of regression equations and theoretical models that are acquired with limited and specific samples and, thus, are not robust in the assessment of hydration outside of the group in which they were developed, and are adversely affected by illness.

The RXc graph (Figure 5) is a probability distribution that classifies a vector according to its distance from the mean healthy vector. The variability of the impedance vector is represented in the bivariate normal distribution with elliptical probability areas (50%, 75%, and 95%) in the tolerance or reference ellipses. Vectors displacements parallel to the major axis indicate changes in hydration (more or less fluids). Vectors within the 50% tolerance ellipse are considered to be at normal hydration whereas lengthening of vectors from the 51% to 75% and >76% percentile of the upper range of percentiles indicate moderate and severe dehydration, respectively. Conversely, shortening of vectors from the 51% to 75% and >76% percentile reference ellipses in the lower range indicate increasing fluid overload. Vectors positioned to the left of the major axis reflect increasing cell mass and vectors to the right indicate decreasing cell mass, respectively. Thus, BIVA uses patterns of impedance vector distribution without the need for prediction equations, body weight or reliance on the assumption of stable composition (water content) of the FFM. BIVA enables detection and ranking of changes in tissue hydration status of <500 mL in real-time [40].

Appropriate derivation and implementation of the RXc graph to classify hydration requires some important technical considerations. All measurements should be acquired with a phase-sensitive (e.g., phase angle directly measured) BI instrument. Failure to use a phase-sensitive device results in significant errors in R (10 Ω) and Xc (10–12 Ω) measurements [41] contributing to 8–10% repositioning of reference and patient vectors. Additionally, use of high-impedance electrodes can lead to misclassification of hydration [42].

### 4.1. Clinical Applications of BIVA

A focal application of BIVA is the assessment of the hydration status in patients with fluid overload and evaluation of effects of therapeutic procedures designed to achieve homeostasis without undesirable side effects. Kidney disease and hemodynamic impairments are the principal conditions in which BIVA is used to classify hydration and to monitor changes in response to treatments. Traditional approaches to assess hydration in these patients are unreliable because they rely on body weight to designate excess fluid and are insensitive to fluid overload in the presence of edema [43]. 

#### 4.1.1. Hydration Assessment in Hemodialysis (HD)

Dialysis, particularly hemodialysis (HD), is amenable to BIVA because of the need for fluid removal and post-dialytic fluid retention. Routine evaluation of hydration includes monitoring of body weight and blood pressure changes that are not reliably determined by fluid volume. Edema is not usually detectable until interstitial fluid volume increases 30% over normal levels (4–5 kg gain in body weight) and severe dehydration can occur before appearance of clinical signs. Thus, traditional indicators of over- and under-hydration in patients with renal disease are insensitive and inadequate [40].

#### 4.1.2. Hydration Changes and Vector Patterns During the HD Cycle

The objective of HD is to remove excess fluid without complications for the patient. Estimation of the volume of fluid for removal is subjective and generally related to previous post-dialytic body weight, which may be unreliable [43]. Fluid removal occurs during HD. It can be symptomatic or uncompensated with episodes of hypotension, malaise, and cramps or asymptomatic (compensated). Fluid overload can occur during the inter-dialytic period and is symptomatic with edema, exacerbated hypertension, and pulmonary congestion. Dry weight is a general term associated with the post-dialytic body weight at which most of the excess fluid has been removed. The optimal “dry weight” is determined clinically and operationally as the weight at which a patient can tolerate HD without adverse intra-dialytic symptoms, notably hypotension. Body fluid volumes, however, change continuously during the inter-dialytic period so that euhydration occurs only for a brief period.

Serial BI measurements and examination of vectors before and after HD reveal a classic pattern of fluid removal and repletion during a standard 3-d HD cycle. Fluid removal via ultrafiltration (UF) accompanies vector lengthening parallel to the mean vector of healthy adults [44]. Within the first 2 post HD, vectors are relatively stable near the post HD vector. During the next 24, 48, and 72 h however, vectors progressively shorten within the 50% to 75% tolerance ellipse and reflect fluid repletion of 1.4, 2.6, and 3.4 L. Thus, BIVA is specific and sensitive to monitor fluid during the wet and dry cycle of HD.

#### 4.1.3. Vector Trajectories: Adequacy of Ultrafiltration (UF)

Measurement of the electrical properties of tissues enables the classification of vectors as abnormal and identification of HD patients at risk of intra-dialytic problems. Observational findings indicate that BIVA can identify potential symptomatic HD patients. Before dialysis, HD patients, compared to healthy controls, exhibit evidence of fluid overload with shorter vectors and lesser phase angles [44,45]. Pre-HD vector position discriminated unstable (symptomatic), compared to stable (asymptomatic), HD patients with significantly longer vectors and smaller phase angles regardless of gender. Vector displacement caused by fluid removal (2.4 L to 2.6 L) further differentiated the HD groups with significantly shorter and less steep vectors among the unstable compared to the stable patients. Patients with vectors cycling within the 75% tolerance ellipse had no symptomatic response during the HD session. However, patients with vectors exceeding the 75% tolerance ellipse (e.g., longer and flatter) were at significantly greater risk for hypotension. This finding provides an operational definition for use of BIVA in HD [46].

Trajectory of an individual vector during UF provides insight into efficacy in HD. Exceeding the 75% tolerance ellipse, longer vectors indicate fluid loss or dehydration (dry vector) and shorter vectors designate fluid overload (wet vector). Vector displacements to the left, compared to the right, of the major axis reveal more versus less cell mass, respectively. A troublesome vector displacement during UF is flat vector displacement to the right associated with an increase in R/H without a proportional increase in Xc/H due to a loss of cells in soft tissue. This pattern is seen in patients with malnutrition or cachexia [45].

### 4.2. BIVA in Peritoneal Dialysis (PD)

Chronic ambulatory peritoneal dialysis (CAPD) is a procedure that performs UF of the lower abdomen by infusion of hypertonic glucose or icodextrin and exchange every 6 hr. The process is continuous rather than cyclical as in HD with the objective to obtain adequate UF and thus achieve normal hydration of the patient. CAPD can result in dehydration or excess fluid removal (decrease in residual urine volume and peritoneal UF) or fluid overload (FO; pitting edema, exacerbation of hypertension, and impairment of UF).

Comparisons of BIVA plots from healthy, HD and CAPD patients reveal differences in tissue electrical properties [47]. Group vectors for pre-HD asymptomatic patients were shorter and flatter (e.g., smaller PhA) within the lower 50% ellipse and lengthened into the 75% ellipse after HD. Whereas mean vectors of non-edematous (compensated) CAPD patients were midway between the vectors of healthy controls and pre-HD values (e.g., overlapping and within the 50% ellipse), it was shorter than the post-HD mean vector. The group vector for edematous (uncompensated) CAPD patients was significantly shorter with smaller PhA in the 95% ellipse. Infusion of dialysate solution (1 L) did not affect R/H, Xc/H, or PhA (0.5%) principally because the thorax only contributes minimally (<10%) to total body Z and thus is relatively insensitive to acute changes in fluid volume. BIVA discriminated FO among the CAPD patients. Specifically, 88% of CAPD patients with pitting edema exceeding the 75% tolerance compared to 12% of non-edematous patients. This observation of a shortened down-sloping vector is consistent with the excess accumulation of interstitial fluid in pitting edema.

Characteristics of an individual vector from a dialysis patient provides an important example of relationships among BI measurements and tissue hydration in relation to FO. Edema, which is an acknowledged sign of FO, is present with increased interstitial pressure due to an expansion of the interstitial fluid volume with concomitant gains in body weight and TBW. In normal hydration, fluid is partitioned between the capillaries and the interstitial compartment that consists of a gelatinous matrix and has a negative interstitial pressure; this condition is characterized with an impedance vector within the 50% tolerance ellipse. Fluid overload is detectable as edema when interstitial pressure changes from negative to positive due to an increase in interstitial fluid volume (30%) and a concomitant gain in body weight (4–5 kg) [48]. Piccoli [47] hypothesized that edema may be expressed with the shortening of the pre-HD vector and displacement in a downward direction toward the lower margin of the 75% tolerance limit corresponding to the increased interstitial fluid pressure. When interstitial fluid pressure increases, most of the fluid increase is free fluid allowing the appearance of pitting edema and bringing the vector outside of the 75% tolerance ellipse, which is defined as the operational threshold for the apparent edema. Fluid overload results in an increase in blood volume so the gain in free fluid in the interstitial space is a release to control blood pressure. Conversely, movement of the vector from the lower to the upper 75% tolerance ellipse indicates a progressive loss of interstitial fluid from the gel meshwork and a decrease in interstitial fluid pressure that reduces blood volume.

### 4.3. BIVA in Critically Ill Patients

Determination of fluid status in the intensive care unit is difficult because of the need for real-time assessments. In the critical care setting, central venous pressure (CVP) measurements are obtained invasively with either a CVP catheter or more invasive hemodynamic monitoring devices to guide fluid infusion. In general, low CVP values designate true or relative hypovolemia and high CVP measurements indicate true or relative hypervolemia or FO. 

BIVA was evaluated as an index of fluid status and compared to CVP measurements in intensive care unit (ICU) patients [49]. Both components of the Z vector (R/H and Xc/H) were significantly, linearly, and inversely correlated with CVP. A progressive increase in CVP matched the shortening and downward displacement of the Z vector out of the lower pole of the 75% tolerance ellipse. Low CVP values were associated with normal or lengthened vectors into the 75% tolerance ellipse, which is indicative of dehydration.

The combination of assessment of peripheral tissue hydration with BIVA and central filling pressure with CVP provides a useful clinical tool for evaluation and planning fluid therapy for patients with low CVP. Specifically, a different response or tolerance to fluid infusion in dehydrated compared to well hydrated patients with the same low CVP, in which BIVA could identify patients with reduced, preserved or increased tissue fluid content.

Among patients admitted to an ICU, BIVA classified 70% as overhydrated [50]. Fluid overloaded patients had a longer stay on the ICU due to persistent fluid retention. Average hydration was a significant (*p* < 0.05) predictor of mortality during the 5 d stay on the ICU (odds ratio (OR) 1.19 (95% CI: 1.04–1.37)) or the 60 d after discharge (OR 1.17, 95% CI: 1.03–1.33).

In a clinician-blinded observational study [51], patients admitted to the ICU were assessed for hydration status with BIVA [52] with concomitant determinations of cumulative fluid balance (input–output) during a 72-h observation period. Patients classified as dehydrated gained 3.4 L whereas over-hydrated patients had a net cumulative fluid balance of –4.5 L. Severe over-hydration was the only significant predictor of mortality on the ICU (OR 22.9, 95% CI: 2.38–2.20; *p* < 0.01).

The presence of acute kidney injury (AKI) in critically ill patients is related to increased mortality with over-hydration, estimated by fluid balance, being an independent contributor to decreased survival [53]. Survivors of AKI in the ICU had longer and steeper group vectors, characterized by greater (*p* < 0.05) R/H and Xc/H values compared to non-survivors diagnosed with AKI [54]. Over-hydration, estimated as mean fluid balance (fluid retention) calculated from diagnosis of AKI until recovery, discharge from ICU, or death, was greater (*p* < 0.001) in the non-survivors compared to the survivors (6.9 L vs. 1.6 L, respectively).

Iatrogenic AKI can occur in patients undergoing coronary angiographic procedures with iodinated contrast media. Prevention strategies include pre-procedural saline infusion to overcome plasma volume depletion. However, identification of patients with low ECF would improve efficacy and outcome of this strategy. Although intravenous volume expansion is a general preventive strategy, a safe, practical, and valid means to identify low hydration status of patients is needed. 

Stable coronary artery disease (CAD) patients with low BIVA-assessed hydration status presented with a significant risk of CI-AKI (contrast-induced acute kidney injury) [55]. Pre-procedural R/H values were greater in patients with CI-AKI than those without CI-AKI (395 Ω/m vs. 352 Ω/m for women, *p* < 0.001; 303 Ω/m vs. 279 Ω/m, *p* < 0.009 for men) indicating lower fluid volume in patients with CI-AKI. Stratification of patients according to R/H values revealed a significantly higher risk of CI-AKI in patients with higher R/H values (OR 2.9, 95% CI: 1.5–5.5; *p* < 0.002). Optimal receiver operating characteristic (ROC) threshold analysis determined R/H values for prediction of CI-AKI risk were 380 Ω/m and 315 Ω/m for women and men, respectively. R/H values exceeding these values were found to be significant and independent predictors of CI-AKI (OR 3.1, 95% CI: 1.8–5.5; *p* < 0.001).

Pre-procedural BIVA determinations were used to classify patients as low or normal hydration status [56]. Based on the previously determined R/H thresholds for the increased risk of CI-AKI [57], patients with low hydration status were randomized to receive a standard (1 mL/kg/h) or double (2 mL/kg/h) saline infusion for 12 h before and after the procedure. The incidence of AKI was significantly lower (11.5% vs. 22.3%; OR 0.45, 95% CI: 0.24–0.85; *p* < 0.015) in the patients receiving double compared to the single saline volume. Thus, evaluation of hydration with BIVA in patients with stable CAD at admission allows identification of hypohydration and adjustment of intravascular fluid volume expansion resulting in lower incidence of CI-AKI after angiography. 

### 4.4. BIVA in Congested Heart Failure (HF)

The pattern of BIVA changes in response to therapy among dialysis patients should be comparable in congestive heart failure patients. Similar to the HD cycle, heart failure is characterized by a cyclical fluid overload (pulmonary and peripheral congestion) and removal (diuretics and extracorporeal fluid removal with dialysis). Despite current therapy, the high rate of readmission emphasizes that the current guidelines for discharge, typically based on clinical impressions, is inadequate for patient stabilization.

Current practice relies on a blood biomarker, N-terminal pro-B-type natriuretic peptide (NT-proBNP), to assess over-hydration in the diagnosis of heart failure (HF). An adaptation of BIVA tolerance ellipses yields the hydration index (HI) that corresponds to soft tissue hydration as hyper- (>74.3%), normo- (72.7% to 74.3%), and hypo-hydration (<72.7%) [57]. Patients diagnosed with HF had significantly greater NT pro-BNP levels, lower R/H and Xc/H values, and greater hydration (81% vs. 74%) than non-HF patients evaluated in the emergency-outpatient department [56]. Massari et al. [58] reported that over-hydration was a significant predictor of the length of stay in hospital (LOS). Patients with a normal hydration index (72.8% to 74.2%) had a shorter LOS (7.36 d (interquartile range or IQR: 7.34 d to 7.39 d); *p* < 0.05)) than other patients classified as over-hydration (9.04 d (IQR: 8.85 to 9.19 d)). Multivariate regression analysis demonstrated that BNP and the hydration index were significant predictors of LOS (*p* < 0.0001). Hydration index and BNP levels were significantly and inversely related. Importantly, peripheral edema was not a predictor of LOS. Thus, congestion determined with BIVA is an important predictor of LOS in HF patients with acute decompensation.

Fluid overload is a risk factor for heart failure patients. Hydration status can be determined from BIVA tolerance ellipses. Nunez et al. [59] classified 369 heart failure patients at discharge and found after 12 months an increased risk of mortality (hazard ratio (HR) 2.08, 95% CI: 1.21–3.58; *p* < 0.008) for over-hydrated patients as well as a linear risk of re-admission of heart failure (HR 1.06, 95% CI: 1.03–1.10; *p* < 0.001).

The combination of admission BNP and discharge BIVA allows prediction of a 90 d mortality risk in HF patients [60]. In a prospective multi-center study, survivors had significantly lower BNP levels (515 pg/L vs. 838 pg/L) and lower hydration (hydration index: 74% vs. 85%), and greater R/H (503.6 Ω/m vs. 445.3 Ω/m) and Xc/H (37 Ω/m vs. 26.7 Ω/m). BIVA was a significant predictor of 90 d mortality (area under the curve (AUC) 0.715, 95% CI: 0.65–0.76; *p* < 0.004)) with Xc/H (AUC 0.712, 95% CI: 0.655–0.76; *p* < 0.007) being a stronger predictor of mortality than R/H (AUC 0.65, 95% CI: 0,29–0.706; *p* < 0.025). Together BNP and BIVA have greater prognostic power for cardiovascular mortality (AUC 0.74, 95%CI: 0.69–0.76; *p* < 0.001).

Dyspnea is a consequence of congestion in HF patients. However, differential diagnosis of acute dyspnea relative to hydration is a challenge. Clinicians blinded to BIVA diagnosed patients presenting with acute dyspnea as cardiac (54%) or non-cardiac etiology based on standard examination criteria. BIVA positions outside the 50% tolerance ellipse (specifically Xc/H) identified peripheral congestion (edema). BIVA measures (vector positions on the RXc plot) accurately discriminated cardiac and non-cardiac dyspnea (69% sensitivity and 79% specificity with 80% AUC analysis) [61]. 

## 5. Bioelectrical Impedance Spectroscopy (BIS)

### BIS in Dialysis

The prediction of fluid volumes with BIS is another approach to classify hydration with an emphasis on the estimation of excess fluid volume [62]. Chamney et al. [63] proposed a model based on body composition that estimates the hydration (fraction, %) of lean and adipose tissue (AT) to estimate FO. They used dual X-ray absorptiometry and tracer dilution and derived fixed values for normal hydration fraction of lean (0.703) and AT (0.197) with different fluid distributions (ECW/ICW, %) in normal lean (0.630) and AT (1.88) to calculate FO, equivalent to excess ECW, from measurements of body weight and BIS-derived estimates of total body ECW and ICW and pre-HD body weight [62]. This approach, termed the physiological tissue model, relies on the assumption of a constant ECW/TBW to identify over-hydration and guide dialysis.

The criteria for the designation of FO are variable and generally dependent on some clinical measures. A pre-HD hydration classification of hydration was a graded FO as normal (−1 L to +1 L), mild (+1 L to <2.5 L), gross (>2.5 L), and under-hydration (−1 L to −2.0 L). The goal of HD was to reach normo-hydration range of fluid excess and remove fluid to reach within 1 L of excess ECW. Application of this model in 500 HD patients revealed only 19% had normo-hydration with systolic BP < 140 mm Hg, 33% had > 2.5 L ECW with BPs < 150 mm Hg, 15% had > 2.5 L ECW, and systolic BP >150 mm Hg [64]. In contrast, 15% patients were hypertensive with FO of 1 L and 10% had systolic BP < 140 mm Hg and FO > 2.5 L. Mean over-hydration was greater (+2.95 L vs. +1.35 L, *p* < 0.05) in patients who died of cardiac causes than patients with non-cardiac deaths [65]. Similarly, significant FO was an independent predictor of survival (HR 1.83, 95% CI: 1.19–2.82, *p* < 0.001)) in peritoneal dialysis (PD) patients [66]. 

An alternative index of hydration is relative fluid overload (RFO) that is the expression of the predicted ECW a percentage of the age- and gender-specific normative ECW [62,63]. Recalculation of RFO from Wabel et al. [67] reveals an RFO of 15% is equivalent to excess ECW > 2.5 L. A follow up study of diabetic HD patients found that gross or excess hydration (RFO > 15%) was associated with an elevated risk of mortality (HR 2.102, 95% CI: 1.389–3.179; *p* < 0.003)) [68]. A few other patients had a decrease in PhA (HR 1.74, 95% CI: 1.37–2.21; *p* < 0.001) independent of age and co-morbidities that predicted mortality of end-stage kidney disease patients [69]. 

Randomized controlled studies revealed that BIS-guided UF resulted in better outcomes for patients whose UF was directed by conventional approaches. Hur et al. [70] found a significant decrease in time-average FO during a one-yr period that lead to significant decreases in myocardial function measures, blood pressure, and antihypertensive medications with BIS-managed UF as compared to control patients. However, patients with BIS-guided UF had a significantly decrease in urine output and an increase in the proportion of patients with anuria. Another study reported decreased mortality (*p* < 0.03) among patients randomly assigned to BIS-predicted UF [71].

Results of large-scale, international, multi-center observational studies of PD patients treated with both PD modalities, CAPD, and automated peritoneal dialysis, reveal important factors related to the estimation of over-hydration using BIS. Unmodifiable patient characteristics (age, male gender, diabetes, and low BMI) and dialysate composition (hypertonic glucose) predicted FO in PD patients in the EuroBCM study [72]. Clinical judgment predicted FO less frequently than BIS in the IPOD-PD study [73]. Inconsistency was observed between the BCM hydration assessment and systolic blood pressure (BP) despite its strong relation to FO [63]. Fifty-six percent of the patients were designated with FO (>1.1 L) of which 24.5% had systolic BP < 140 mm Hg whereas 38.7% were normo-hydrated and 28.2% had systolic BP > 140 mm Hg, and among 4.9% classified as dehydrated, 3.4% had elevated systolic BP. Among the PD patients assessed as normally hydrated by clinical judgment, >30% appeared FO with BIS. These findings indicate a limitation of BIS to adequately detect fluid in the trunk, which is consistent with the inability of the applied current to enter the peritoneum and the relatively low contribution of the trunk to total body BI measurements [12,74]. 

Consensus on the ability of BIS to detect sequestered fluid in the peritoneum is lacking. Siphai et al. [75] measured FO before and after removal of peritoneal dialysate and found a non-significant 240 mL difference. Parmentier et al. [76] similarly reported a negligible (50 mL) difference in FO estimated before and after a PD session. Arroyo et al. [77], however, found that FO measured after an infusion of dialysate was greater (180 mL; *p* < 0.043) than after a removal of dialysate, and concluded that BIS prediction of FO should be performed after drainage of the abdomen. Age, severity of end-stage renal disease (ESRD), nutritional status, and different sample sizes affect the interpretation of the results. However, the modest differences in apparent FO confirm the limitation of BIS to reliably detect fluid in the truck and specifically the abdomen.

Recent findings indicate no benefit of BIS-guided fluid management among non-anuric PD patients on residual renal function (RRF) and cardiovascular function. Oh et al. [78] studied 137 adults who had no differences in age, gender ratio, cause of kidney failure, duration of PD, glomerular filtration rate (GFR), or peritoneal transport type for one year. Use of BIS resulted in no differences in net changes in estimated over-hydration or ECW/TBW, echocardiographic parameters, or arterial stiffness. 

Tabinor and Davies [79] conclude that the usefulness of BIS to guide fluid management in dialysis is not well defined. They remark that BIS can monitor fluid change but the need is to link BIS-estimated fluid changes with intermediate variables (blood pressure, cardiac dynamics, and morphology) with a goal to preserve residual kidney function; evidence of relationships with mortality is lacking. Additional research with more coordinated and consistent research designs is needed.

## 6. BIVA in Nutrition Assessment

Patients with kidney disease are progressively at risk for impaired nutritional status, exacerbation of renal function and protein energy wasting. Surveys reveal both suboptimal intakes of macro- and micro-nutrients with increased losses of micronutrients associated with metabolic acidosis, hormonal dysregulation, and inflammation that increase mortality risk in patients with chronic kidney disease (CKD) and can lead to ESRD [80,81]. Protein energy wasting is acknowledged to cause muscle loss and potentially precipitate intra-dialytic complications [63].

Identification of altered body composition with BIVA comes from early observation of CKD patients referred for treatment. Compared to healthy, age-, and gender-matched adults, CKD patients had a shorter and downward sloping mean vector at the lower boundary of the 75% tolerance ellipse [82]. Patients had significantly decreased R/H, Xc/H, and PhA values. PhA decreased in relation to the severity of CKD. Furthermore, diabetic CKD patients had the shortest and most downward vector displacements. Whereas differences in PhA globally indicate proportional losses of body cell mass, provided that hydration is considered [83], they importantly reflect differences in the fluid distribution. Specifically, a decrease in PhA designates an increase in ECW/ICW due to disproportionate loss of muscle and thus an indication of fluid overload. 

BIVA distinguishes fluid excess and nutritional status in uremic HD patients. Under-nutrition assessed with the subjective global assessment (SGA), significantly affected vector displacements before and after an HD session [84]. Before dialysis, the mean vector of HD patients classified with normal nutritional status (SGA-A) was within the 50% tolerance ellipse. In contrast, the mean vectors of the patients with moderate and severe malnutrition (SGA-B and C) decreased into the 75% and outside the 95% tolerance ellipses, respectively, indicating progressive fluid accumulation and excess. Importantly, the contributions of Xc progressively decreased (A < B < C). Fluid removal (2400 mL) resulted in a lengthening of the mean vectors due to large changes in R/H for the normal nutrition and moderately malnourished patients, indicating fluid removal, and similar changes in Xc/H. In contrast, the mean vector of the severely malnourished patients flattened with a smaller change in R/H associated with less fluid removal (1800 mL), due to complication during the dialytic session, and a negligible change in Xh/H consistent with reduced body cell mass. Thus, BIVA discriminates fluid excess from the nutritional status and identifies deficit in muscle mass in malnourished HD patients. 

## 7. Equivalence of Bioimpedance Measurements

There is a paucity of data comparing BIVA and BIS patterns in the same subjects. Piccoli et al. [16] performed simultaneous phase-sensitive 50 kHz BI and phase-sensitive BIS measurements on renal patients before and during an HD session. A pattern of congruence between groups vectors on the RXc graph and the peak of each Cole plot (Fc or critical frequency) emerged indicating equivalence of information from these two BI approaches. Specifically, the Cole plots progressively enlarged and moved to the right on the RXc graph during the 180 min dialytic session with Fc nearly identical to the Xc at 50 kHz so that the Z vector tracked dialytic fluid removal similarly as the Cole plots. The Xc values at 5 kHz, which were expected to be 0 Ω if the conduction was only extracellular, were 70% of the maximum (Fc) indicating intracellular current flow at low frequencies and suggesting anisotropy. Additionally, correlation coefficients relating R and Xc at 50 kHz with all other frequencies were 0.96 to 0.99 and 0.65 to 0.99, respectively, demonstrating an equivalence of BI measurements. They observed similar findings in a comparison of the same BI instruments in body builders and non-resistance training men [85]. Notably, the ratio of Xc values at 5 kHz to Fc were 70% and 64% for the body builders and control men, respectively, indicating a substantial intracellular flow of current in both groups. The R and Xc measurements at 50 kHz were highly correlated with the same measurements at frequencies ranging from 0 to infinity and ranged from 0.94 to 1.0. Thus, there was equivalence of information provided by the vector components of R and Xc at 50 kHz compared with the measurements from BIS. This equivalence persists with fluid removal and expanded ICW with increased muscularity.

An important question is the equivalence of BI measurements obtained from various impedance instruments in the same subjects. Phase angle measured in patients before an HD session with a phase-sensitive BIS compared to a phase-sensitive 50 kHz device was significantly less (4.2° vs. 4.7°, respectively) with no difference in R (515 Ω vs. 510 Ω, respectively). This difference in PhA values indicates that BIS can underestimate Xc presumably as a result of the mathematical modeling of the Td factor as well as anisotropy in tissues [86]. These findings reaffirm previous observations that BI instruments do not provide similar values of R, Xc, and PhA and conclude that BI devices should not be used interchangeably [83].

### Standardization of BI Measurements

Small differences in BI measurements can adversely impact direct and indirect assessments of hydration. Kyle et al. [9] provided the systematic discussion of factors to enable reliable BI measurements of adults. Gonzalez-Correa and Calcdeo-Eraso [87] provided some estimates of error for factors that moderate BI measurements. Notable errors include inspiration (5%), electrode placement (1 cm = 3%), and increased core (2 °C) and skin (33.5 °C) temperature (8%) that are important because technical reproducibility is ~2% [9]. Importantly, disease conditions, compared to healthy matched adults, exerted large effects on BI measurements up to 35%, which indicates the ability of BI to identify fluid disturbances.

Although reliability of BI measurements tends to be high [9,83], Genton et al. [88] reported significant differences in PhA values between various BI instruments (50 kHz phase-sensitive and BIS). Silva et al. [89] identified small but significant differences in BI measurements between 50 kHz, phase-sensitive BI and multi-frequency BIS devices. Compared to the single-frequency, phase-sensitive BI, the BIS device significantly underestimated R (9.9 Ω; limits of agreement (LOA) = −40 Ω to 21 Ω) and Xc (0.97 Ω; LOA = −6 Ω to 4 Ω) and overestimated PhA (0.12°; LOA = −0.4° to 0.5°).

Another confounding variable is the use of inappropriate surface electrodes. Whereas some BI device manufacturers specify the appropriate electrodes based on physical dimensions, the literature reveals diversity in commercial electrodes used with the same BI instrument. Nescolarde et al. [42] found large differences in R and Xc measured in nine commercial pre-gelled silver-silver chloride adhesive electrodes with wide variations in inherent R (11 Ω to 665 Ω) and Xc (0.25 Ω to 2.5 Ω) that significantly affected vector position on the RXc graph resulting in significant differences in hydration classification of healthy adults.

Standardization of BI measurements remains a challenge for clinical users of BI for hydration classification. The lack of international manufacturing standards, coordination of technology, and cross-calibration of electrical accuracy hinders growth in clinical applications of BI. Concurrence on appropriate measurements conditions in hydration assessment is imminent but clinical investigators need to be aware of elevated core and peripheral temperatures as well as high impact BI measurements. Use of artifact-free contact electrodes is recommended for reliable classification of hydration. Small differences in BI measurements incurred by measurement influences can alter hydration classification in clinical conditions.

## 8. Summary and Conclusions

Bioimpedance measurements are gaining interest to assess and monitor hydration status in dialytic and critically ill patients because they overcome many of the limitations of traditional methods of hydration assessment. Whereas two approaches use of BI measurements each has specific advantages and certain limitations.

Use of direct 50 kHz, phase-sensitive BI measurements in the BIVA model for hydration assessment is progressing in a variety of clinical conditions. BIVA illustrates a vector whose position is evaluated relative to healthy reference ranges and is interpreted as normal or abnormal hydration based on distance from the mean healthy vector. Migration of the vector (shortening or lengthening) in response to progression of a physiological process, pathology, or an intervention indicates changes in hydration (gain or loss of fluids). Classification of hydration status (normal or abnormal) avoids insensitivities (>10% variability and imprecision for individual estimations) associated with regression equations and unproven biophysical models, and does not rely on body weight to assess hydration. Use of BIVA improves the prescription of UF in dialysis by monitoring the backward-forward displacement of vectors in relation to the wet-dry cycle of HD. It further enhances decision-making in dialysis by facilitating the interpretation of alterations in blood pressure relative to the hydration status and, thus, adjusting UF. Among critically ill patients, BIVA is significantly and inversely correlated with CVP. On the RXc graph, impedance vectors of patients with low, normal, and high CVP move downward and outside of the 75% tolerance level with increasing CVP, which indicates excess fluid accumulation. Identification of hypohydration in stable CAD patients before angiographic procedures enabled rehydration with appropriate volumes of saline to attenuate risk of CI-AKI. Use of BIVA in assessment of malnutrition, notably in illnesses with muscle loss when hydration is altered, is increasing.

Phase-sensitive multi-frequency BIS measurements coupled with biophysical models indirectly estimate ECW that is compared with projections of normal ECW to calculate excess ECW described as FO. The estimates of ECW, adjusted for BMI, are derived from regression equations using gender, height, and body weight as independent variables with wide limits of agreement and, hence imprecision for an individual ECW estimate. Estimates of FO >2.5 L or >15% predicted ECW are associated with increased morbidity in dialysis patients but the prognostic value for mortality is lacking. 

## 9. Future Directions

Advancement of the use of BI measurements to aid in fluid management of dialysis patients requires prospective controlled studies with more focus on outcomes [79,90,91,92]. Early efforts were descriptive emphasizing proof-of-principle research. Adequate confirmation of positive findings related to validity BIVA and BIS is available. New research should critically evaluate the benefit of these BI approaches in patient care. Importantly, there is a need for more consistency and scrutiny in research design with appropriate sample sizes to test hypotheses and comparability in reporting of results. Some topics include the ability to predict intra- and inter-dialytic complications and mortality of HD patients, measure the effects of therapeutic interventions on changes in hydration and fluid status on preservation of renal function, evaluate the specificity and sensitivity of available and putative clinical markers of hydration, determination of changes in body composition as well as nutritional status/intake relative to longevity of dialysis, and the effect of interventions to preserve muscle mass on survival. The goal is to provide findings that can be applied practically to improve patient outcomes.
